# Conservative Management of Patent Ductus Arteriosus Is Feasible in the Peri-Viable Infants at 22–25 Gestational Weeks

**DOI:** 10.3390/biomedicines11010078

**Published:** 2022-12-28

**Authors:** Misun Yang, Yun Sil Chang, So Yoon Ahn, Se In Sung, Heui Seung Jo, Won Soon Park

**Affiliations:** 1 Department of Pediatrics, Samsung Medical Center, Sungkyunkwan University School of Medicine, Seoul 06351, Republic of Korea; 2 Cell and Gene Therapy Institute, Samsung Medical Center, Seoul 06351, Republic of Korea; 3 Department of Health Sciences and Technology, SAIHST, Sungkyunkwan University, Seoul 06351, Republic of Korea; 4 Department of Pediatrics, Kangwon National University Hospital, Kangwon National University School of Medicine, Kangwon 24289, Republic of Korea; 5 Department of Pediatrics, CHA Gangnam Medical Center, CHA University, 566 Nonhyeon-ro, Gangnam-gu, Seoul 06135, Republic of Korea

**Keywords:** patent ductus arteriosus, preterm infant, conservative management, fluid restriction

## Abstract

The purpose of this study was to determine the natural course of hemodynamically significant (HS) patent ductus arteriosus (PDA) with conservative management and whether the presence or prolonged duration of HS PDA affected mortality/morbidities in infants at 22–25 weeks estimated gestational age (EGA). We retrospectively reviewed the medical records of 77 infants born at 22–25 weeks EGA, stratified into 22–23 weeks (*n* = 21) and 24–25 weeks EGA (*n* = 56). HS PDA was present in 77%, 76%, and 77%, and open ductus at discharge was 12%, 13%, and 12% in the total and at 22–23 and 24–25 weeks EGA infants, respectively. For backup rescue treatment, 7% and 5% of the infants received oral ibuprofen and device closure, respectively. A mortality rate of 9% was found in the HS PDA (+) infants, significantly lower than the 28% in HS PDA (−) infants. There are no significant differences in morbidities. In multivariate analyses, the presence and/or prolonged duration of HS PDA was not associated with increased mortality or morbidity. Spontaneous closure of HS PDA was achieved through conservative management in the peri-viable infants at 22–25 weeks EGA.

## 1. Introduction

Recent advances in perinatal and neonatal intensive care have improved the survival of peri-viable infants at 22–25 weeks estimated gestational age (EGA) [1,2,3,4,5]. However, the optimal management of hemodynamically significant (HS) patent ductus arteriosus (PDA) in infants at the highest risk of development remains contentious [1,6,7,8]. Due to the high failure rate of pharmacologic treatment, ductal ligation was performed in up to 47–64% of this high-risk population [3,6]. In contrast, since PDA was considered a normal physiological finding in infants at 22–24 weeks EGA at a Swedish center [1], the rule was the expectant waiting without routine initial echocardiographic screening [9], and PDA mostly closed spontaneously [10], requiring only 5–10% late surgical ligation of PDA [1]. Although these findings suggest that the conservative management of PDA can be successfully implemented even in peri-viable infants at 22–25 weeks EGA, the pros and cons of the conservative management of PDA in these infants remain unclear due to a paucity of published data.

Previously, we reported that exclusive conservative management of HS PDA with few backup treatments was safe and feasible in extremely preterm infants [11,12,13,14]. Furthermore, the exclusive conservative management of all PDAs, regardless of clinical and echocardiographic severities, with limited backup rescue treatments in extremely preterm infants, has become a standard treatment in our neonatal intensive care unit (NICU) since 2012. In the present study, we focused on the natural course of HS PDA in peri-viable infants at 22–25 weeks EGA receiving conservative management with few backup rescue treatments. The secondary aim of this study was to determine whether the presence and/or prolonged duration of HS PDA in peri-viable infants had adverse effects on the risk of mortality and/or morbidities.

## 2. Materials and Methods

### 2.1. Ethics Statement

The data collection procedure was approved by the Institutional Review Board (IRB) at the Samsung Medical Center (SMC) (IRB No. SMC 2022-08-011). The IRB waived the requirements for informed consent due to the retrospective design of this study. All the methods were performed in accordance with the relevant guidelines and regulations.

### 2.2. Study Population and Follow-Up Protocol for HS PDA

We retrospectively reviewed the medical records of 77 inborn infants (birth weight ≥ 400 g) at 22–25 weeks EGA without major congenital anomalies admitted to and discharged from the SMC neonatal intensive care unit (NICU) between 1 January 2020, and 31 March 2022, with available initial echocardiogram data obtained within the first two postnatal weeks. The details of the 16 infants excluded from the analysis are summarized in Figure S1. We stratified the infants into 22–23 and 24–25 weeks EGA to better identify any weeks EGA dependent variations in mortality, major morbidities, and the incidence and natural course of HS PDA observed in the previous studies [1,2,3,4,5,6,11,12,13,14], and compared the mortality and morbidities such as bronchopulmonary dysplasia (BPD), necrotizing enterocolitis (NEC), and intraventricular hemorrhage (IVH) according to the presence or absence and prolonged duration of HS PDA. HS PDA was defined as ductal diameter ≥ 1.5 mm and left atrium (LA)/aorta (Ao) ratio ≥ 1.5, with predominant left-to-right shunt on echocardiography (GE Vivid E90; Horten, Norway) initially performed within the first two postnatal weeks in ventilated infants with signs suggestive of hemodynamically significant PDA, including cardiac murmur, hypotension, widened pulse pressure, or respiratory deterioration. HS PDA (−) was defined as ductal diameter < 1.5 mm or LA/Ao ratio < 1.5 on echocardiography, with no requirement of mechanical ventilator support, regardless of PDA size. Two-dimensional, M-mode, and color flow Doppler imaging were performed for ductal diameter and LA/Ao ratio measurements by cardiology fellows or trained sonographers, and the results were confirmed by the attending cardiologists. Despite the high chances of missing capture of the infants with a silent PDA, follow-up echocardiography was conducted if any signs suggestive of HS PDA occurred, occasionally or regularly, at 2–6-week intervals until PDA closure or discharge from the NICU.

### 2.3. Conservative Management Protocol of HS PDA

Throughout the study period, all infants with PDA were exclusively managed with conservative treatment, with few backup rescue treatments. Judicious fluid restriction was initiated at birth and maintained for the first two months of life. After administering an initial fluid volume of approximately 70 mL/kg/day on postnatal day (P)1, each infant’s daily target fluid volume was adjusted after clinically evaluating the volume status, including the body weight and electrolyte changes. The PRN diuretic therapy was started with furosemide (1 mg/kg/dose) for infants with decreased urine flow < 1–2 mL/kg/h with signs of fluid retention such as skin edema, cardiomegaly, or pulmonary congestion. When the infants had cardiopulmonary compromise such as congestive heart failure, increased ventilator setting with progressing pulmonary congestion in chest radiography, and blood laboratory abnormalities such as metabolic acidosis, hypercapnia, or hyponatremia despite conservative management of HS PDA including judicious fluid restriction and use of inotropics and/or diuretics, the attending neonatologists assessed and determined the need for backup rescue treatments after cardiac consultation [11].

### 2.4. Data Collection

The clinical characteristics, including the gestational age (GA), birth weight, Apgar scores at 1 and 5 min, sex, delivery mode, small for gestational age (SGA; birth weight below the 10th percentile), antenatal steroid use, and chorioamnionitis were analyzed. The GA was determined from the obstetric findings based on the last menstrual period and/or first-trimester ultrasonography and postnatal modified Ballard test. Chorioamnionitis was confirmed based on the placental pathology. Diuretic and inotropic use was defined as the use of diuretics for >2 consecutive days and the use of dopamine and/or dobutamine for >1 day within the first 4 weeks of life, respectively.

We investigated the short-term outcomes, including death before discharge, BPD (grade ≥ 2, defined by Jensen et al.) [15], IVH (grade ≥ 3) [16], NEC (Bell’s stage ≥ 2b) [17], retinopathy of prematurity requiring laser treatment [18], acute kidney injury (AKI; KDIGO stage 3) [19], and sepsis (defined as positive blood culture in symptomatic infants plus > 5 days of antibiotic treatment after postnatal days 7).

The cumulative incidence rates of ductal patency at 22–23 and 24–25 weeks EGA were analyzed to evaluate the natural course of HS PDA according to the GA. We use multivariate regression analyses to calculate the adjusted odds ratios for the mortality and morbidities of BPD, IVH, NEC, ROP, and AKI with 95% confidence intervals according to the presence and/or prolonged duration (per week) of HS PDA.

### 2.5. Statistical Analysis

Continuous variables were expressed as the mean (standard deviation) and compared using the Student’s *t*-test, while the Mann–Whitney U test was conducted for group sample sizes less than 30. Categorical variables were expressed as percentages and frequencies and compared using the chi-square test or Fisher’s exact test. The cumulative incidence rates of PDA were demonstrated according to the GA using the Kaplan–Meier estimation. Multivariable analyses using binary logistic regression were performed to calculate the adjusted odds ratios for the risk of adverse outcomes according to the presence and prolonged (per week) duration of HS PDA. SPSS Version 26.0 (SPSS Inc., Chicago, IL, USA) was used for all statistical analyses, and *p* < 0.05 was considered statistically significant.

## 3. Results

### 3.1. Prevalence and Natural Course of HS PDA with Conservative Management

**Figure 1:**
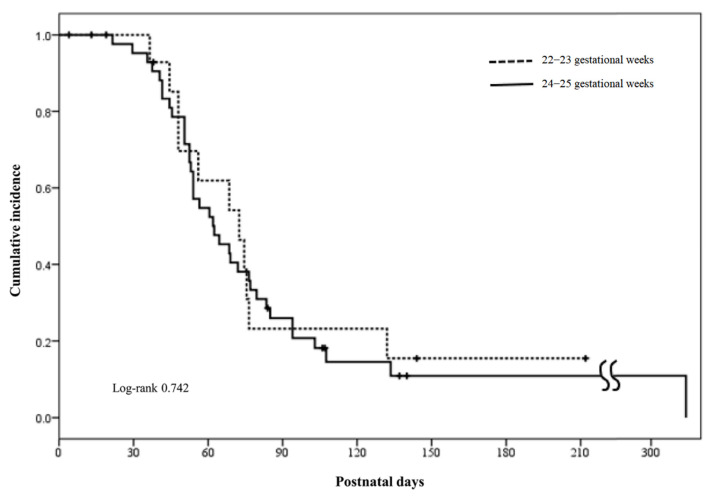
Cumulative incidence of ductal patency during hospitalization in the infants with initial HS PDA according to gestational weeks.

The prevalence rates of HS PDA were 77% (59/77), 76% (16/21), and 77% (43/56) in the total population and infants at 22–23 and 24–25 weeks EGA, respectively (Figure S1). While all infants received exclusive conservative management regardless of HS PDA, significantly larger ductal size and LA/Ao ratio at first diagnosis and later closure of PDA were observed in the HS PDA (+) group compared with the HS PDA (−) group (Table 1). However, no significant differences were observed in the mean days of PDA closure and similar 13% (2/16) vs. 12% (5/43) rates of open ductus at discharge between the HS PDA (+) subgroups at 22–23 and 24–25 weeks EGA (Figure 1, Table 1). In addition, while no infant received surgical ligation, 7% (4/59) received backup oral ibuprofen treatment, and 5% (3/59) of the infants in the HS PDA (+) group underwent device closure of PDA immediately before discharge or during outpatient follow-ups.

### 3.2. Clinical Characteristics, Fluid and Energy Intake

The clinical characteristics and fluid and energy intakes with or without HS PDA in the 22–23 and 24–25 weeks EGA subgroups are shown in Table 2 and Table 3. While the Apgar score at 1 min, cesarean delivery, and diuretic use in the total HS PDA (+) group were significantly higher than the HS PDA (−) group, no significant differences were found in the Apgar score at 5 min, antenatal steroid, and inotropic use between the HS PDA (+) and (−) groups. However, in the subgroup analysis, significantly lower inotropic use was observed in the 24–25 weeks EGA compared to the 22–23 weeks EGA subgroup, regardless of HS PDA (Table 2).

The initial mean fluid intake at P1 of 71–75 mL/kg/day gradually increased to 123–127 mL/kg/day at P28. Fluid intake included intravenous fluid volume and enteral feeding volume. Enteral feeding was started tropically on P1, gradually increased according to the infant’s condition, and full enteral feeding was achieved at the average of 56 days with wide individual variation (standard deviation 33 days). No significant differences were observed in the fluid and energy intake from P1 to P28 between the HS PDA (+) and (−) groups (Table 3). The serum sodium levels were in the normal range despite fluid restriction from P1 to P28 between the study groups (Table S1). Provision of adequate caloric intake for anabolism and the resultant adequate weight gain after P7 and similar weight gain at discharge were achieved when compared to other units where conservative fluid management was not used [3].

### 3.3. Adverse Outcomes

The prevalence of adverse outcomes, including death before discharge and BPD, is summarized in Table 4. Death in the HS PDA (−) group (4/13, 31%) was significantly higher than the HS PDA (+) group (2/43, 5%) at 24–25 weeks EGA, and the cause of death was NEC with multi-organ failure, pulmonary hypertensive crisis with severe BPD and respiratory failure due to underlying lung hypoplasia in 2, 1 and 1 infants, respectively. While the duration of invasive ventilation in the 22–23 weeks EGA was significantly higher than 24–25 weeks EGA regardless of HS PDA (Table S2), no significant differences in other adverse outcomes, including BPD, were observed between the HS PDA (+) and HS PDA (−) groups (Table 4).

### 3.4. Adjusted Odds Ratios for Risk of Adverse Outcomes

In multivariate analyses, the adjusted odds ratios for the risk of adverse outcomes such as death before discharge and major morbidities, including BPD were not significantly increased by HS PDA or prolonged duration of HS PDA (Table 5).

## 4. Discussion

Appropriate management of HS PDA in peri-viable infants at 22–25 weeks EGA remains an ongoing challenge [6]. Watkins et al. [3] reported a high rate (72–75%) of PDA in infants at 22–25 weeks EGA, and the GA-dependent high failure rate of medical treatment [20,21,22] resulted in 47–64% of these infants receiving ductal ligation. In contrast, while our data showed a comparably high 77% prevalence of HS PDA as reported by Watkins et al. [3], with ≥moderate clinical (≥C3) showing and echocardiographic criteria (≥E3) showing [23] in the infants at 22–25 weeks EGA, the HS PDA was successfully managed with conservative management. The PDA remained open only in 12% of these infants at NICU discharge. Four infants (7%) received backup oral ibuprofen rescue treatment, and device closure was conducted in three infants (5%) owing to cardiopulmonary compromise refractory to conservative management. Still, none of the infants underwent surgical ligation of the PDA. Overall, despite single center experience and small sample size, our data suggest that conservative management with few backup treatments could be successfully implemented for the most severe HS PDA, and spontaneous closure of PDA could be mostly achieved with conservative management in peri-viable infants at 22–25 weeks EGA [1,11,12,13,14]. A further well-designed prospective larger multi-center trial would be necessary to clarify this.

Despite the lack of proof of their direct cause-and-effect relationship, significant associations between the presence [24,25,26,27] and prolonged duration of HS PDA [27,28] and increased risk of mortality/morbidities were observed in some studies [13,14]. Our data of increased mortality observed in the HS (−) PDA than HS (+) at 24–25 weeks EGA were due to complications of extreme prematurity, including NEC and septic shock not related to HS PDA. In our present and previous retrospective studies, the presence and prolonged duration of both clinically and echocardiographically severe HS PDA was not associated with increased risks of mortality and/or morbidities. Moreover, in our double-blind prospective randomized clinical trial [11], a significantly higher HS PDA closure rate at 1 week after randomization was observed with oral ibuprofen (34%) than with conservative management (7%) in the infants in the 27–30 weeks EGA subgroup, but not the 23–26 weeks EGA subgroup. However, the incidence of death or BPD was similar between the two study groups. In concordance with our data, Clyman et al. [29] reported that moderate-to-large PDAs in infants at <28 weeks EGA were associated with an increased risk of BPD/death only when the infants required intubation for ≥10 days. Collectively, these findings suggest that the apparent association between the presence and prolonged duration of HS PDA might be primarily attributable to immaturity itself, and conservative management of HS PDA with few backup rescue treatments might be feasible in peri-viable infants at 22–25 weeks EGA.

Considering the high cumulative prevalence of stage 3 AKI (57–72%) during the first few weeks of life in infants at 23–26 weeks EGA [19], which indicates low glomerular filtration due to immature nephrogenesis, the success of conservative management for HS PDA might be dependent on maintaining low fluid intake goals to avoid fluid overload during the first two months of life [11,12,13,14]. High fluid intakes >170 mL/kg/day in the first week of life was associated with an increased risk of PDA [30] and BPD/death [31]. Moreover, in contrast to our data showing 12% of open ductus in infants at 22–25 weeks EGA with judicious fluid restriction, Semberova et al. [32] reported that 32% of the infants at <26 weeks EGA who received liberal fluid intake were discharged home with open ductus. Taken together, these findings suggest that meticulous fluid therapy to avoid excessive fluid intake along with adequate energy intake, initiated at birth, might be a prerequisite for successful conservative PDA management, thereby reducing the prevalence of HS PDA, enhancing its earlier closure, and reducing any associated mortality and/or morbidities [33].

This study had several limitations, including a single-center, retrospective, and uncontrolled design. In addition, a small number of the HS PDA (−) subgroup of infants at 22–23 weeks EGA (*n* = 5) might be the biggest drawback of this study and have contributed to the under-power of this study for showing statistically significant differences between the study groups. Nonetheless, the relatively large sample size of 77 infants at 22–25 weeks EGA born and admitted to a single center over a relatively short time period with similar baseline characteristics and the clinical management policy used to diagnose and treat HS PDA of these peri-viable infants might be strong aspects of this study. Although we strictly identified HS PDA (defined as PDA size ≥ 1.5 mm plus LA/Ao ratio ≥ 1.5 on echocardiography only in intubated and mechanically ventilated infants with clinical deterioration) to avoid the possibility of selection bias favoring infants with lower disease severity [27,34], the arbitrary criteria for HS PDA and thereby the lack of robust echocardiographic markers to estimate shunt volumes might be another major limitation of this study. Developing more detailed and accurate definitions of HS PDA, including the volume status of the left heart, would be necessary for better identifying high-risk infants who might benefit from therapeutic intervention with more accuracy. In addition, wide and heterogeneous follow-up echocardiography intervals (every 2–6 weeks) might not have allowed for accurate comparisons of the timing of spontaneous closure of PDA between the study groups.

In conclusion, conservative management of HS PDA with few backup rescue treatments was successfully implemented, and the PDA predominantly closed spontaneously in peri-viable infants at 22–25 weeks EGA. Furthermore, the presence and prolonged duration of HS PDA were not associated with increased mortality and/or morbidities, including BPD, in these infants. These findings warrant well-designed prospective controlled studies in the future to clarify further the advantages and disadvantages of exclusive conservative management of HS PDA in infants born at 22–25 weeks EGA.

## Figures and Tables

**Table 1:** PDA-associated variables and outcomes of the infants with or without HS PDA according to gestational weeks.

	22–23 Gestational Weeks			24–25 Gestational Weeks			Total	
	HS PDA (−) (*n* = 5)	HS PDA (+) (*n* = 16)	Total (*n* = 21)	HS PDA (−) (*n* = 13)	HS PDA (+) (*n* = 43)	Total (*n* = 56)	HS PDA (−) (*n* = 18)	HS PDA (+) (*n* = 59)
First diagnosis of PDA								
Age at first diagnosis, days	6.8 (3.3)	7.3 (3.6)	7.2 (3.5)	7.4 (3.9)	6.4 (1.7)	6.7 (2.4)	7.2 (3.6)	6.7 (2.4)
PDA size at first diagnosis, mm	2.1 (0.8)	2.4 (0.5)	2.3 (0.6)	1.9 (0.7)	2.6 (0.5) ^a^	2.5 (0.6)	2.0 (0.7)	2.5 (0.5) ^a^
LA/Ao ratio at first diagnosis	1.4 (0.3)	1.8 (0.3)	1.7 (0.3)	1.2 (0.2)	1.8 (0.3) ^a^	1.8 (0.3)	1.3 (0.2)	1.8 (0.3) ^a^
Age at ductal closure, days	43.2 (16.3)	66.6 (26.0)	61.6 (25.7)	35.9 (26.4)	69.5 (47.4) ^a^	58.9 (25.4)	37.6 (24.0)	68.8 (43.2) ^a^
Backup treatment								
Oral ibuprofen, *n* (%)	0 (0)	0 (0)	0 (0)	0 (0)	4 (9)	4(7.1)	0 (0)	4 (7)
Age at first ibuprofen treatment, days					58.8 (17.2)			
Device closure, *n* (%)	0 (0)	1 (6)	1 (5)	0 (0)	0 (0)	0 (0)	0 (0)	1 (2)
Age at device closure, months		4						
Surgical ligation, *n* (%)	0 (0)	0 (0)	0 (0)	0 (0)	0 (0.0)	0 (0)	0 (0)	0 (0)
PDA open until discharge								
*n* (%)	1 (20)	2 (13)	3 (14)	0 (0)	5 (12)	5 (9)	1 (6)	7 (12)
Spontaneous closure at OPD, *n* (%)	1 (20)	0 (0)	1 (5)	0 (0)	0 (0)	0 (0)	1 (6)	0 (0)
Age at spontaneous closure, months	17							
Device closure at OPD, *n* (%)	0 (0)	1 (6)	1 (5)	0 (0.0)	1 (2)	1 (2)	0 (0.0)	2 (3)
Age at device closure, months		17			9			

Values are presented as means (standard deviations) or *n* (%). PDA, patent ductus arteriosus; HS, hemodynamically significant; LA, left atrium; Ao, Aorta; OPD, outpatient department. ^a^: *p* < 0.05 compared with HS PDA (−).

**Table 2:** Clinical characteristics of the infants with or without HS PDA according to gestational weeks.

	22–23 Gestational Weeks			24–25 Gestational Weeks			Total	
	HS PDA (−) (*n* = 5)	HS PDA (+) (*n* = 16)	Total (*n* = 21)	HS PDA (−) (*n* = 13)	HS PDA (+) (*n* = 43)	Total (*n* = 56)	HS PDA (−) (*n* = 18)	HS PDA (+) (*n* = 59)
Baseline variables								
GA, weeks	23.0 ± 0.9	23.5 ± 0.3	23.4 ± 0.5	24.9 ± 0.7 ^b^	25.0 ± 0.7 ^b^	25.0 ± 0.7 ^b^	24.4 ± 1.1	24.6 ± 0.9
Birth weight, g	548 ± 79	597 ± 84	585 ± 83	658 ± 155	725 ± 143 ^b^	710 ± 147 ^b^	627 ± 145	691 ± 141
Male sex	2 (40)	7 (44)	9 (43)	8 (62)	22 (51)	30 (54)	10 (56)	29 (49)
Apgar score, 1 min	3.6 ± 1.1	5.6 ± 0.8 ^a^	5.1 ± 1.2	4.5 ± 5.0	5.0 ± 1.6	4.9 ± 1.6	4.2 ± 1.6	5.2 ± 1.5 ^a^
Apgar score, 5 min	7.2 ± 0.8	7.6 ± 0.8	7.5 ± 0.8	7.4 ± 1.1	7.6 ± 1.4	7.5 ± 1.3	7.3 ± 1.0	7.6 ± 1.2
Cesarean delivery	3 (60)	15 (94)	18 (86)	10 (77)	39 (91)	49 (88)	13 (72)	54 (92) ^a^
SGA	0 (0)	2 (13)	2 (10)	4 (31)	7 (16)	11 (20)	4 (22)	9 (15)
Multiple pregnancy	3 (60)	7 (44)	10 (48)	5 (39)	23 (54)	28 (50)	8 (44)	30 (51)
Antenatal steroid	5 (100)	13 (82)	18 (86)	12 (92)	42 (98)	54 (96)	17 (94)	55 (93)
Maternal hypertension	0 (0)	1 (6)	1 (5)	2 (15)	5 (12)	7 (13)	2 (11)	6 (10)
Oligohydramnios	1 (20)	4 (25)	5 (24)	3 (23)	5 (12)	8 (14)	4 (22)	9 (15)
Chorioamnionitis	4 (80)	11 (69)	15 (71)	7 (54)	22 (51)	29 (52)	11 (61)	33 (56)
PROM	2 (40)	5 (31)	7 (33)	4 (31)	8 (19)	12 (21)	6 (33)	13 (22)

Values are presented as means ± standard deviations or *n* (%). HS PDA, hemodynamically significant patent ductus arteriosus; GA, gestational age; SGA, small for gestational age; PROM, premature rupture of membrane. ^a^: *p* < 0.05 compared with HS PDA (−). ^b^: *p* < 0.05 compared with the infants at 22–23 gestational weeks.

**Table 3:** Fluid and energy intake of the infants with or without HS PDA according to gestational weeks.

	GA 22–23 Weeks			GA 24–25 Weeks			Total	
	HS PDA (−) (*n* = 5)	HS PDA (+) (*n* = 16)	Total (*n* = 21)	HS PDA (−) (*n* = 13)	HS PDA (+) (*n* = 43)	Total (*n* = 56)	HS PDA (−) (*n* = 18)	HS PDA (+) (*n* = 59)
Fluid intake								
Input at P1	76.6 ± 4.1	75.0 ± 4.7	75.3 ± 4.5	71.7 ± 8.3	73.5 ± 5.6	73.1 ± 6.3 ^b^	73.0 ± 7.6	73.9 ± 5.3
Input at P7	135.7 ± 5.5	135.3 ± 9.6	135.4 ± 8.7	137.7 ± 9.8	125.1 ± 14.2 ^a,b^	128.0 ± 14.3 ^b^	137.1 ± 8.7	127.9 ± 13.8 ^a^
Input at P14	132.6 ± 13.4	128.0 ± 13.6	129.2 ±13.3	127.2 ± 18.4	120.9 ± 14.3	122.2 ± 15.2	128.9 ± 16.8	122.7 ± 14.3
Input at P21	120.1 ± 22.7	128.5 ± 13.1	126.3 ± 15.9	129.3 ± 10.8	122.9 ± 15.4	124.2 ± 14.8	126.5 ± 15.3	124.3 ± 15.0
Input at P28	124.4 ± 15.5	126.7 ±14.4	126.1 ± 14.3	129.2 ± 12.0	122.7 ± 15.2	124.1 ± 14.7	127.7 ± 12.9	123.7 ± 15.0
Calorie intake								
Calorie at P1	37.0 ± 5.3	36.1 ± 5.6	36.3 ± 5.4	36.2 ± 7.0	35.5 ± 6.3	35.7 ± 6.4	36.4 ± 6.4	35.7 ± 6.1
Calorie at P7	61.7 ± 6.7	73.2 ± 12.6	70.5 ± 12.4	85.1 ± 17.1 ^b^	78.8 ± 12.7	80.2 ± 14.0 ^b^	78.6 ± 18.3	77.3 ± 12.8
Calorie at P14	73.9 ± 19.8	84.3 ± 16.4	81.7 ± 17.4	86.3 ± 18.9	83.3 ± 13.3	83.9 ± 14.5	82.4 ± 19.4	83.5 ± 14.0
Calorie at P21	74.3 ± 12.2	93.0 ± 21.8	88.1 ± 21.2	83.7 ± 14.2	91.7 ± 14.5	90.0 ± 14.6	80.7 ± 13.9	92.0 ± 16.4 ^a^
Calorie at P28	84.8 ± 19.1	95.6 ± 17.9	92.7 ± 18.3	89.3 ± 10.5	94.3 ± 13.4	93.2 ± 12.9	87.9 ± 13.3	94.6 ± 14.5

Values are presented as means ± standard deviations or *n* (%). HS PDA, hemodynamically significant patent ductus arteriosus; *p*, postnatal day. ^a^: *p* < 0.05 compared with HS PDA (−). ^b^: *p* < 0.05 compared with the infants at 22–23 gestational weeks.

**Table 4:** Adverse outcomes of the infants with or without HS PDA according to gestational weeks.

	22–23 Gestational Weeks			24–25 Gestational Weeks			Total	
	HS PDA (−) (*n* = 5)	HS PDA (+) (*n* = 16)	Total (*n* = 21)	HS PDA (−) (*n* = 13)	HS PDA (+) (*n* = 43)	Total (*n* = 56)	HS PDA (−) (*n* = 18)	HS PDA (+) (*n* = 59)
BPD (grade ≥ 2) or death	3 (60)	6 (38)	9 (43)	6 (46)	10 (23)	16 (29)	9 (50)	16 (27)
Death before discharge	1 (20)	3 (19)	4 (19)	4 (31)	2 (5) ^a^	6 (11)	5 (28)	5 (9) ^a^
BPD (grade ≥ 2)	2/4 (50)	3/13 (23)	5/17 (29)	3/10 (30)	9/42 (21)	12/52 (23)	5/14 (36)	12/55 (22)
NEC (stage ≥ IIb)	2 (40)	3 (19)	5 (24)	3 (23)	10 (23)	13 (23)	5 (28)	13 (22)
IVH (grade ≥ III)	0 (0)	2 (13)	2 (10)	1 (8)	3 (7)	4 (7)	1 (6)	5 (9)
ROP (laser treatment)	2/4 (50)	6/13 (46)	8/17 (47)	3/10 (30)	11/42 (26)	14/52 (27)	5/14 (36)	17/55 (31)
AKI (KDIGO stage 3)	1 (20)	6 (38)	7 (33)	7 (54)	15 (35)	15 (35)	8 (44)	21 (36)

Values are presented as *n* (%). HS PDA, hemodynamically significant patent ductus arteriosus; BPD, bronchopulmonary dysplasia; NEC, necrotizing enterocolitis; IVH, intraventricular hemorrhage; ROP, retinopathy of prematurity; AKI, acute kidney injury. ^a^: *p* < 0.05 compared with HS PDA.

**Table 5:** Adjusted odds ratios for the risk of adverse outcomes by presence or duration of HS PDA.

	22–23 Gestational Weeks		24–25 Gestational Weeks		Total	
	Adjusted OR (95% CI)	*p*-Value	Adjusted OR (95% CI)	*p*-Value	Adjusted OR (95% CI)	*p*-Value
Risk of adverse outcomes by presence of HS PDA						
BPD (grade ≥ 2) or death	0.22 (0.01–7.89)	0.41	0.38 (0.09–1.61)	0.19	0.37 (0.13–1.10)	0.08
Death before discharge	0.22 (0.00–16.07)	0.49	0.14 (0.02–1.00)	0.05	0.29 (0.06–1.48)	0.14
BPD (grade ≥ 2)	0.45 (0.01–22.28)	0.69	0.67 (0.12–3.83)	0.66	0.47 (0.11–1.98)	0.30
NEC (stage ≥ IIb)	0.46 (0.01–22.86)	0.70	1.27 (0.25–6.43)	0.77	1.11 (0.28–4.38)	0.89
IVH (grade ≥ III)	-		0.93 (0.07–12.45)	0.96	1.17 (0.11–12.57)	0.90
ROP (laser treatment)	2.08 (0.05–82.30)	0.70	0.68 (0.13–3.71)	0.66	0.70 (0.17–2.85)	0.61
AKI (KDIGO stage 3)	-		0.36 (0.08–1.58)	0.18	0.72 (0.21–2.53)	0.61
Risk of adverse outcomes by prolonged duration (per week) of HS PDA						
BPD (grade ≥ 2) or death	1.11 (0.79–1.58)	0.55	0.95 (0.71–1.28)	0.74	1.01 (0.84–1.23)	0.88
Death before discharge	0.31 (0.04–2.22)	0.24	-		0.66 (0.40–1.09)	0.10
BPD (grade ≥ 2)	-		0.97 (0.72–1.31)	0.84	1.12 (0.91–1.37)	0.31
NEC (stage ≥ IIb)	2.81 (0.51–15.44)	0.24	1.00 (0.71–1.39)	1.00	1.06 (0.85–1.32)	0.62
IVH (grade ≥ III)	-		2.25 (0.97–5.24)	0.06	1.06 (0.81–1.39)	0.69
ROP (laser treatment)	1.03 (0.57–1.86)	0.93	1.00 (0.76–1.33)	0.98	1.01 (0.84–1.23)	0.89
AKI (KDIGO stage 3)	1.05 (0.74–1.49)	0.79	1.03 (0.77–1.37)	0.87	0.98 (0.81–1.18)	0.83

Multivariate logistic regression test adjusted for gestational age, birth weight, cesarean delivery, and Apgar score at 1 min. HS PDA, hemodynamically significant patent ductus arteriosus; OR, odds ratio; CI: confidence interval; BPD, bronchopulmonary dysplasia; NEC, necrotizing enterocolitis; IVH, intraventricular hemorrhage; ROP, retinopathy of prematurity; AKI, acute kidney injury.

## Data Availability

All data generated or analyzed during this study are included in this article. Further enquiries can be directed to the corresponding author.

## Supplementary Materials

The following supporting information can be downloaded at: https://www.mdpi.com/article/10.3390/biomedicines11010078/s1, Figure S1: Study population of the infants with or without HS PDA according to gestational weeks. Table S1: Sodium level in blood and body weight gain of the infants with or without HS PDA according to gestational weeks. Table S2: Comparison of duration of respiratory support with or without HS PDA according to gestational weeks.
