# Supplementary material for: Conservative Management of Patent Ductus Arteriosus Is Feasible in the Peri-Viable Infants at 22–25 Gestational Weeks
Source: Biomedicines. 2022 Dec 28;11(1):78. doi: 10.3390/biomedicines11010078 (PMC9855634; doi:10.3390/biomedicines11010078)
Supplement: Supplementary file 1 [file biomedicines-11-00078-s001.zip › biomedicines-2127556-supplementary.pdf]

Figure S1. Study population of the infants with or without HS PDA according to gestational weeks.

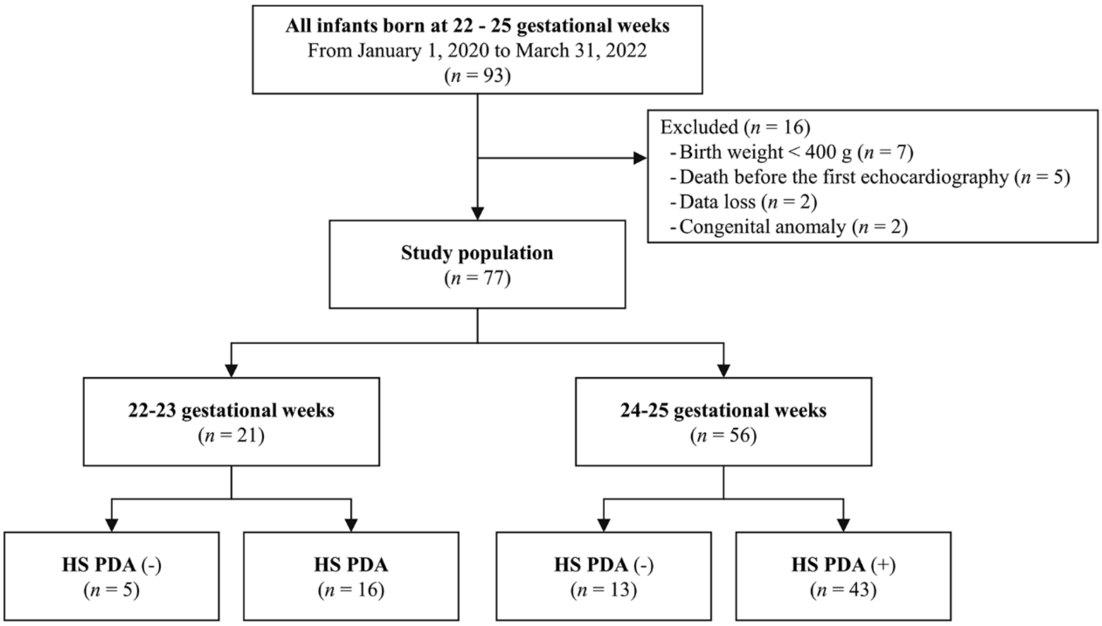

HS PDA; hemodynamically significant patent ductus arteriosus

Table S1. Sodium level in blood and body weight gain of the infants with or without HS PDA according to gestational weeks

	GA 22-23 weeks			GA 24-25 weeks			Total	
	**HS PDA (-) (*n* = 5)**	**HS PDA (+) (*n* = 16)**	**Total (*n* = 21)**	**HS PDA (-) (*n* = 13)**	**HS PDA (+) (*n* = 43)**	**Total (*n* = 56)**	**HS PDA (-) (*n* = 18)**	**HS PDA(+) (*n* = 59)**
Na level (meq/L)								
Na at P1	139.6 ± 4.0	**140.8 ±1.7**	**140.5±2.4**	138.2±2.2	**137.2±3.2**[b]	**137.4±3.0**[b]	138.6±2.7	138.9±5.6
Na at P7	**139.2 ± 4.6**	**135.0 ± 3.1**[a]	136.0±3.8	138.8±6.1	135.0±5.9	135.9±6.1	**138.9±5.6**	**135.0±5.3**[a]
Na at P14	139.6 ± 3.0	**140.1 ± 3.7**	**140.0±3.5**	138.7±3.1	**137.5±5.1**[b]	**137.7±4.8**[b]	139.0±3.0	138.2±4.9
Na at P21	138.0 ± 3.7	136.3 ± 2.9	136.7±3.1	136.6±6.2	135.8±4.5	135.9±4.8	137.0±5.5	135.9±4.1
Na at P28	136.6 ± 7.7	134.4 ± 4.2	135.0±5.2	137.1±4.5	135.1±4.4	135.6±4.5	136.9±5.4	135.0±4.3
Body weight gain (g/kg/day)								
First week	-6.1 ± 7.4	-5.0 ± 8.2	-5.3 ± 7.8	-4.5 ± 9.4	-6.3 ± 8.2	-5.9 ± 8.4	-5.0 ± 8.7	-5.9 ± 8.1
Second week	9.8 ± 17.9	18.8 ± 9.6	16.6 ± 12.3	17.2 ± 7.8	16.6 ± 13.2	16.7 ± 12.3	14.9 ± 11.8	17.2 ± 12.4
Third week	20.6 ± 21.0	15.4 ± 11.4	16.8 ± 14.0	18.1 ± 12.1	13.7 ± 11.3	14.6 ± 11.5	18.9 ± 14.7	14.1 ± 11.2
Fourth week	18.7 ± 16.5	14.8 ± 12.1	15.8 ± 13.0	18.1 ± 7.7	16.4 ± 8.1	16.7 ± 8.0	18.3 ± 10.6	16.0 ± 9.2
Body weight (g)								
Corrected age of 40 weeks or discharge	3013 ± 592	3056 ± 533	3049 ± 524	2681 ± 545	2956 ± 490	2903 ± 507	2758 ± 550	2980 ± 497

Values are presented as means ± standard deviations or *n* (%). HS PDA, hemodynamically significant patent ductus arteriosus; P, postnatal day.

[a]: $p < 0.05$ compared with HS PDA (-). [b]: $p < 0.05$ compared with the infants at 22-23 gestational weeks.

Table S2. Comparison of duration of respiratory support between with or without HS PDA according to gestational weeks.

	**GA 22-23 weeks**			**GA 24-25 weeks**			**Total**	
	**HS PDA (-) (*n* = 5)**	**HS PDA (+) (*n* = 16)**	**Total (*n* = 21)**	**HS PDA (-) (*n* = 13)**	**HS PDA (+) (*n* = 43)**	**Total (*n* = 56)**	**HS PDA (-) (*n* = 18)**	**HS PDA(+) (*n* = 59)**
Duration of invasive ventilator								
Mean±SD (days)	61±3	81±86	76±75	57±71	51±48	52±53	58±59	58±60
Median (IQ) (days)	62(58-63)	59(45-73)	**61(49-71)**	27(18-73)	44(30-62)	**43(26-67)**[b]	51(23-65)	47(31-47)
Duration of non-invasive ventilator								
Mean±SD (days)	46±18	25±19	30±21	42±25	31±18	33±19	**43±22**	**30±18**[a]
Median (IQ) (days)	43(30-64)	19(8-42)	27(13-45)	40(35-47)	28(18-41)	29(19-42)	**40(35-48)**	**27(17-41)**[a]
Duration of both invasive and non-invasive ventilator								
Mean±SD (days)	107±16	106±83	106±72	103±63	82±44	86±48	104±52	85±56
Median (IQ) (days)	105(93-122)	86(64-115)	88(67-118)	73(47-129)	73(63-90)	73(63-95)	101(68-122)	74(63-91)
Duration of oxygen application								
Mean±SD (days)	26±6	37±27	**35±25**	20±15	23±18	**23±18**[b]	22±13	27±22
Median (IQ) (days)	26	29 (19-61)	**28 (20-44)**	18(9-33)	19(12-32)	**19(11-31)**[b]	20(10-30)	21(13-32)

[a]: $p < 0.05$ compared with HS PDA (-). [b]: $p < 0.05$ compared with the infants at 22-23 gestational weeks.

SD, standard deviation; IQ, interquatertile range.
